# Recent Advances and Future Direction in Lyophilisation and Desiccation of Mesenchymal Stem Cells

**DOI:** 10.1155/2016/3604203

**Published:** 2016-08-14

**Authors:** Akalabya Bissoyi, Awanish Kumar, Albert A. Rizvanov, Alexander Nesmelov, Oleg Gusev, Pradeep Kumar Patra, Arindam Bit

**Affiliations:** ^1^Department of Biomedical Engineering, National Institute of Technology, Raipur 492010, India; ^2^Department of Biotechnology, National Institute of Technology, Raipur 492010, India; ^3^Institute of Fundamental Medicine and Biology, Kazan Federal University, Kazan 420000, Russia; ^4^Preventive Medicine & Diagnosis Innovation Program (PMI), Division of Genomic Technologies, RIKEN, Yokohama 2300045, Japan; ^5^Department of Biochemistry, Pt. JNM Medical College, Raipur 492001, India

## Abstract

Mesenchymal Stem Cells (MSCs) are a promising mammalian cell type as they can be used for the reconstruction of human tissues and organs. MSCs are shown to form bone, cartilage, fat, and muscle-like cells under specific cultivation conditions. Current technology of MSCs cryopreservation has significant disadvantages. Alternative technologies of mammalian cells preservation through lyophilisation or desiccation (air-drying) are among the upcoming domains of investigation in the field of cryobiology. Different protectants and their combinations were studied in this context. Loading of the protectant in the live cell can be a challenging issue but recent studies have shown encouraging results. This paper deals with a review of the protectants, methods of their delivery, and physical boundary conditions adopted for the desiccation and lyophilisation of mammalian cells, including MSCs. A hybrid technique combining both methods is also proposed as a promising way of MSCs dry preservation.

## 1. Introduction

Mesenchymal Stem Cells (MSCs) are multipotent stromal cells able to differentiate into different cell types, including chondrocytes, adipocytes, and osteoblasts [1–3]. MSCs have potential utility in cell therapy and regenerative medicine, with applications relating to tissue engineering and as vehicles for gene therapy. Advantages of their usage include high plasticity, regenerative, and immunosuppressive properties and tropisms toward inflamed, hypoxic, and cancerous sites [2]. Additionally, the typical usage of MSCs for the patient was derived from autologous transplantation. It is biologically safe and free of any ethical issues associated with the source of cell.

The usage of MSCs requires their preservation in a way allowing them to be rapidly available for an application. The commonly used approach for MSCs storage is cryopreservation using liquid nitrogen [4]. This technology can ensure a high viability of stored cells but the storage, transfer, and prevention of contamination are complicated and relatively expensive. Lyophilisation (freeze-drying) and desiccation (air-drying) combined with the application of specific protective compounds emerge as a promising strategies for the storage of mammalian cells, including MSCs. As these technologies allow the storage of dried samples at ambient temperature, they can greatly simplify storage and distribution of samples, thereby decreasing the preservation cost. However, the usage of these technologies requires a revision of existing protocols in order to enhance the cell viability rates. For this purpose, we need an improved understanding of processes underlying the cell survival during water loss and in the dry state.

This paper discusses the fundamental principles, mechanisms, and advantages of the lyophilisation and desiccation usage for MSCs preservation. It also briefly describes an application and possible crosstalk of these technologies, usage of different lyoprotectants, and physiological factors involved in the cell response.

## 2. Lyophilisation and Desiccation

**Figure 1:**
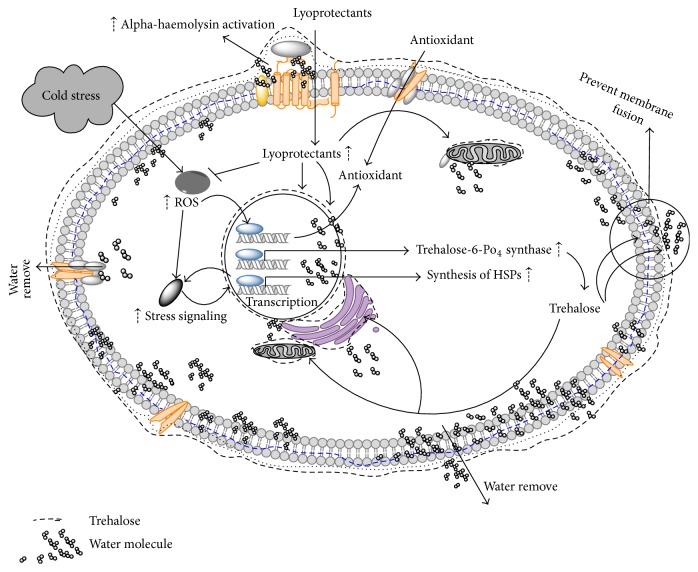
Molecular mechanics involved in cell lyophilisation.

Lyophilisation is a process of drying of the frozen sample when frozen water is removed in two steps: primary drying (sublimation), followed by secondary drying (desorption). Since lyophilisation is performed at low temperature, it is used to stabilize and prolong shelf life of thermolabile products and those products which are otherwise unstable in aqueous state and hence need to be dried. The principle involved is sublimation of water at temperature and pressure below its triple point, that is, 611 Pascal and 0.0098 degree Celsius. Sublimation takes place after the sample is frozen by supplying heat through conduction or radiation or both. The driving force behind the sublimation or removal of water is the water vapour concentration gradient between the drying front and the condenser [5]. Similarly to the cryopreservation, different poly-hydroxy compounds such as sugars, polyalcohols, and their derivatives can be used in lyophilisation to protect the products that are sensitive to occurring dehydration. These compounds are called lyoprotectants and are reviewed below. The diagram summarizing the processes beyond the cell lyophilisation in the presence of nonreducing disaccharide trehalose is depicted on (Figure 1). Lyophilisation was reported to ensure the viability up to 70% of MSCs without addition of protectants [6].

Desiccation is a nature-driven process, widely used by plants and lower animals as a way to survive the water deficiency. In response to desiccation, such organisms switch to the specific state of suspended animation, anhydrobiosis, allowing them to revive when water returns [7]. Organisms capable of anhydrobiosis were found among a variety of taxa, including bacteria, yeast, invertebrates, and plants [8]. The common process underlying anhydrobiosis is an accumulation of sugars such as trehalose or sucrose [7]. In the dry state these sugars form a highly viscous glass-like medium that has been shown to be necessary for stabilization of cell components [9]. In addition, anhydrobiotes were shown to utilize a variety of protective proteins such as LEA (Late Embryogenesis Abandoned) and heat-shock proteins (HSPs), proteins of antioxidant system, or transport proteins [10]. Successful short-term storage of human fibroblast was achieved [11], but successful MSCs desiccation is not reported yet.

## 3. Protectants in Lyophilisation and Desiccation

A variety of protectants were tried for the dry preservation of mammalian cells. Among them, nonreducing disaccharides trehalose and sucrose are the most popular because they are used by almost every anhydrobiotic animal. There are two main hypotheses explaining the protective effect of sugars in desiccation: (i) water-replacement hypothesis and (ii) vitrification hypothesis [9]. The water-replacement hypothesis proposes that sugars replace water interacting with polar or charged groups of biomolecules via direct hydrogen bonds. Thereby sugars stabilize the native structure of phospholipid membranes or proteins in the absence of water. The vitrification hypothesis assumes that sugars ensure a glass-like state of both cytoplasm and extracellular medium, preventing denaturation or mechanical disruption of membranes and cellular components. Proteins, such as LEA, can also play an important role in the formation of vitrified medium during desiccation [9]. Protectants naturally used by anhydrobiotic animals were successfully used for lyophilisation.

Nonreducing sugars were preferred as protectants for desiccated objects since they are less reactive than reducing monosaccharides or disaccharides like maltose. Maltose has proven to be an effective lyoprotectant for DNA [12], but its higher reactivity should be taken into account for the dry storage. Similarly, nonreducing disaccharide sucrose was shown to be more susceptible to hydrolysis into reducing monosaccharides than trehalose [7]. This indicates that trehalose might be a preferable over sucrose as a protectant in applications requiring the storage of dry samples. Interestingly, widely used cryoprotectants such as dimethyl sulfoxide (DMSO) or glycerol did not find a usage as protectants during desiccation, due to fundamental differences between these modes of preservation.

## 4. Advantages of Trehalose over Other Protectants

Trehalose is widely assumed as the protectant superior to others in the desiccation context. The hydration radius of trehalose is 2.5 times greater than that of sucrose, which implies that 2.5 times the concentration of sucrose is required to provide the same protein protection properties as trehalose [13]. Trehalose interacts more strongly with both water and proteins [14] and is able to displace water molecules which are bound to carbonyls at the phospholipid bilayer of cell membranes unlike to sucrose [15]. Trehalose has higher glass transition temperature (*T*_*g*_) than sucrose which means that when a small amount of water is added, *T*_*g*_ for sucrose goes below the storage temperature and hence the sample dried with sucrose degrades more rapidly as compared to trehalose dried product (whose *T*_*g*_ remains above storage temperature). In addition, antioxidant effect of trehalose was shown [16]. Hence, due to all these properties of trehalose, it can be considered to be superior to the sucrose as an effective protectant for the dry storage (Table 1).

## 5. Synergistic Enhancers

Different combinations of protective agents were tried for the desiccation of cells, since it is clear from existing studies that anhydrobiotic animals typically use the interplay of several mechanisms. Similarly to the case of trehalose, these combinations were mostly inspired by their naturally existing prototypes in different anhydrobiotic organisms. Study shows that more than 40 mM trehalose could effectively maintain the viability of MSC more than 92% for 7 days at 4°C storage temperature [17].

Expression of small heat-shock proteins (HSPs) is tightly linked to the stress tolerance, including the desiccation tolerance in African chironomid* Polypedilum vanderplanki* [18]. Probably, chaperone activity of these proteins can mitigate deleterious effects of water loss. Transfection of embryonic cells of human kidney with the gene Hsp26 protein coupled with trehalose application exhibits a sharp rise in survival after desiccation in comparison to trehalose alone [19]. This improved cell survival clearly demonstrates a useful synergy of trehalose and HSP for the recovery of cells undergoing desiccation and rehydration. Expression level of Hsp26 protein can be further increased in order to improve this method of cell drying [19].

Another promising way to improve desiccation tolerance of cells is the use of LEA proteins. LEA proteins stabilize vitrified sugar glasses which are important in the dried state [20] and protect desiccation-liable proteins from deactivation and aggregation [21]. Additionally, LEA proteins were shown to interact with biological membranes [22]; protein studied in this work was only afterwards identified as LEA protein [23]. Recently, LEA protein from African chironomid* Polypedilum vanderplanki* was reported to ensure the same level of protection as intracellular trehalose, as judged by membrane integrity after rehydration [24]. Since existing LEA proteins differ in their characteristics and localization [24, 25], their combination with each other or with other protectants is a promising way of engineering of cells tolerant to desiccation.

Combination of trehalose with other molecules has also been tested for an increase in cell viability compared to usage of trehalose alone. A study involving desiccation of bovine sperm using sorbitol as a substitute for trehalose proves that in bovine sperm during dehydration it can be used as an excellent osmolyte and it also offers improvement in motility of sperm during desiccation. Sorbitol was found to be enhanced protection on permeating through the plasma membrane, whose mechanism is yet to understand properly [26]. Glycerol also has a significant role in combination with trehalose in achieving higher postrehydration membrane integrity in the desiccation of adipose tissue derived adult stem cells [27].

Recent studies involve the components of stress alleviating pathways such as chelators. For instance, sperm exhibited improved desiccation tolerance and enhanced survival rates when they were coupled with either sucrose or intracellular trehalose along with 1 mM Desferal to the desiccation buffer. Although sperm motility at postdrying stage did not show significant improvement, an increase in membrane integrity of sperm was observed [28]. Furthermore, recent study shows that sucrose, trehalose, and raffinose pretreatment improve postthaw viability of MSCs after cryopreservation [29].

Synergistic effect of trehalose and Arbutin (antioxidant) associated with an induction of HSPs in human MSCs was also shown [30]. These findings may highlight the correlation between the enhancer molecules and desiccation tolerance in mammalian cells which should be taken into account to derive a standard protocol.

## 6. Trehalose Delivery

Presence of protectants such as trehalose on both sides of the cell membrane is important for adequate protection of intracellular components and cell membrane. However, the key protectants trehalose and sucrose are not membrane permeable. In typical case, MSCs do not have transporters for those protectants. The possible use of native membrane pores such as P2X7 [35] is very limited because they are specific to only a few cell types. Human MSCs are also capable of loading trehalose from the extracellular space by a fluid-phase endocytosis [34] but this process is inefficient and cell type dependent [37]. Thus the presence of trehalose in the MSCs should be achieved artificially.

The number of proteins were shown to transport trehalose or other protective sugars into the cells, such as MalEFGK2, MAL11/AGT1, or TRET1 [38]. The advantages of TRET1 transporter are its high-capacity, specificity to the trehalose, neutral pH optimum, and expression from one gene [38]. Stable expression of TRET1 in Chinese hamster ovary (CHO) cells causes an astonishing increase by 170% of the cell viability after desiccation [38]. However, the use of this transporter may not be suitable for the MSCs preservation since this approach requires the genetic modification of the cells. The same reason may block the use of trehalose synthesis inside the cells despite of successful expression of trehalose producing genes in human fibroblast cells [32].

Direct methods of trehalose loading have also been used for the delivery of trehalose. Electropermeabilization was first tried as a trehalose delivery mechanism by Shirakashi et al. in mouse myeloma cells in isotonic trehalose substituted medium [31]. Microinjection was also successfully used for the trehalose loading in human oocytes, an attempt to improve their cryosurvival [39]. However, the poration of the cell membrane allows nonspecific transport of molecules and ions. Resulting alteration of transmembrane ionic balance may lead to significant cell damage.

A novel technique of trehalose loading involves the use of basic amino acid rich cell penetrating peptides (CPPs), such as peptide KRKRWHW [39]. This peptide was designed to deliver trehalose into mammalian cells on the base of molecular simulations. Trehalose, as a cargo coupled with the KRKRWHW peptide through hydrogen bond and *π*–*π* bond, was successfully loaded into the Mouse Embryonic Fibroblasts (MEFs) [36]. This CPP is able to efficiently deliver trehalose into mammalian cells with low cytotoxicity even at high concentrations. Therefore, this CPP may be very helpful for improving the tolerance of cells during desiccation. Similarly, Toner and his group used a genetically engineered mutant of* Staphylococcus aureus* *α*-hemolysin to create pores and to load trehalose into the cytoplasm of fibroblast cells [33]. In this study, trehalose was used as cryoprotectant.

Recently, a successful trehalose loading into cells was achieved through modification of its molecule [32]. Intracellular concentration of trehalose was sufficient for applications in biopreservation while the impact on cellular viability and function was negligible. Different ways of trehalose delivery into mammalian cells are summarized in Table 2.

## 7. Role of Physical Factors and Cell Culture Condition

The physical conditions of the macroenvironment and microenvironment of cells play a major role in the desiccation tolerance of desiccated cells. The beneficial aspect of this approach is an elimination of genetic engineering of the cells. A thorough study of the effects of various physical conditions should be carried out to make conclusions for optimization of favourable limits for all the physical parameters. Primarily, vacuum conditions improve the cell tolerance to desiccation in case of lyophilisation compared to air-drying. In case of MSCs, lyophilisation can ensure the cell viability up to 70% even without trehalose [6]. However, results of this study cannot be directly extrapolated to the cell storage because the cell viability was studied only at 2 h after lyophilisation.

Electromagnetic cryopreservation is undergoing a lot of research as a way of cryopreservation of MSCs, since static and oscillating electric fields and magnetic fields influence the ice formation [40]. Dental pulp stem cells preserved in a programmed freezer using magnetic field show around 70% viability when recovered, suggesting that magnetic freezing may be an alternate method for MSC preservation [41].

We have studied also the effect of drying rate on cell viability. Cultured cells were dried in tissue culture plates in the airflow of a biosafety cabinet. The drastically increased cell-death was observed in an absence of trehalose (data not shown). Ceasing laminar airflow caused a reduction of the rate of desiccation and an increase of cell recovery. Interestingly, clearly different cell viability was observed among different wells and within different areas of the same well. A better cell survival was observed at the well periphery, whereas reverse occurred at the centre of the well (data not shown).

In a different set of experiments we demonstrated that MSC-like cells from third molar tooth germs maintained their biological properties, including expression of MSC surface antigens CD29, CD73, CD90, CD105, and CD166, expression of pluripotency associated genes, proliferation, and differentiation ability [42]. Importantly, cryopreserved cells displayed neuroprotective effects in a model of neuroblastoma SH-SY5Y cells exposed to oxidative and chemotherapeutic stress conditions.

Light causes deleterious effect on the cells during desiccation, probably because of the increase of the free radicals concentration. Fluorescence light was shown to generate free radicals within hamster of human cells, thereby facilitating oxidative DNA lesion and single-strand breaks. Therefore, cells desiccated and maintained at dark condition have greater viability than those exposed to the fluorescence light.

In order to optimize the ability of cell culture to withstand desiccation, the effects of desiccation temperature and cell culture confluency were also investigated. Optimal desiccation tolerance was found at higher cell density. Cell aggregation decreases their tolerance to desiccation. The temperature of operation for cell desiccation greatly influences the viability of cells, and the optimal survival point was found to be slightly lower than room temperature (20°). Recovery of desiccated cells was sufficient at this optimum temperature only. Therefore, it can be summarized that vacuum encapsulates the cells during the process of desiccation thereby improving the cell viability. Also, it had been found that cells can survive without addition of carbohydrates (or polyols) if the slow process of desiccation is followed by vacuum storage. A consistent outcome of the procedure can be obtained by defining a desiccation process which preserves cellular structures. Even in this case, gradual loss of viability may be found because of destruction with time of the desiccated state due to release of free radicals [11].

## 8. Application of Lyophilisation and Desiccation and Molecular Mechanism Involved

Survival of cells in dry and desiccated states is few of the most fascinating phenomena of nature and is lot to explore. The ability of MSCs to withstand these conditions for sustaining high degree of viability could have applications for wide spectrum of biological sciences, including tissue engineering. From above discussion it can be concluded that the lyophilisation and desiccation are two competing formulators for long-term storage of MSC. Both these technologies have been quite successfully applied in a wide range of fields including tissue engineering. Preservation of MSCs through desiccation in the dry state is used to meet the growing demand for MSCs storage and transport in regenerative medicine. Desiccation provides significant advantages over lyophilisation because of simplicity and higher energy efficiency. However desiccation may cause cell stress such as changes in cell volume, osmotic pressure, shrinkage of membrane/cell organelles, changes in activity of enzyme, metabolism downregulation, increased salt concentrations, cell viscosity changes, and stress protein production [43]. Lyophilisation seems to be more safe technology in this respect since it helps to avoid damage maintaining sample frozen throughout drying. Lower cell damage and loss of activity and higher degree of dehydration are the principle advantages of lyophilisation. Lyophilisation should be used when complete rehydration needed and product are heat liable, unstable, or high valuable. However, lyophilisation may also cause problems due to changes in liquid phase, solubility, and water freezing [44]. Thus, both processes have their own pros and cons and different application.

The potential of MSCs in regenerative medicine increases the importance of development of optimized protocols for their desiccation and lyophilisation. Uptake of protectants by MSCs is required for successful desiccation or lyophilisation. However, uptake of protectant by cells can cause cellular response specific to the cell type. Since HSPs are able to improve the cell viability and protect cells from apoptosis during freeze-drying [19], some MSCs cells may be more resistant because of their ability to produce higher HSP levels. Similarly, MSCs can differ in antioxidant response to the activation of stress sensing mechanisms under desiccation or lyophilisation. Since antioxidants have protective potential for a dry storage [30], this difference should also be taken into account.

## 9. Crosstalk between the Process of Lyophilisation and Desiccation

**Figure 2:**
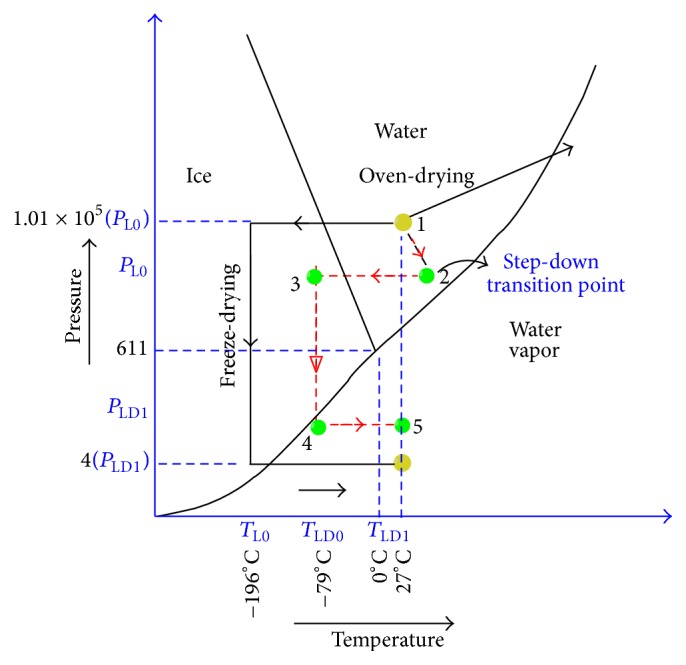
Phase diagram for cells under lyophilisation and desiccation.

Thermodynamic aspects of the above two processes of preservation of MSC are interestingly competitive. Each process can involve quantization of temperature and pressure for transition from one state to another in order to improve cellular viability.  Figure 2 shows a typical thermodynamic diagram for fluidic matter (specifically extracellular and intracellular fluid).

In the figure, black arrowed path indicates the normal process of lyophilisation which involves vitrification followed by creation of extensive vacuum at a temperature of −79 degrees centigrade. This process is expensive as well as time consuming. On the other hand, desiccation involves greater cellular mortality due to rapid dehydration of cellular masses by overshooting the base temperature of the cellular medium to a higher point. A brief description of the heat transfer accounted during the process of lyophilisation is described below.

Bioheat equation for heat transfer in a system of suspended cells in ultrathin straw (UTS) undergoing for slow cooling or vitrification can be realised by zero-dimensional heat transfer equation and it is given as

(1) $$\begin{array}{c} \frac{1}{r}\frac{\partial }{\partial r}\left(\;\lambda r\frac{\partial T}{\partial r}\;\right)+\frac{\partial }{\partial z}\left(\;\lambda \frac{\partial T}{\partial z}\;\right)+\dot{q}=\rho c_{p}\frac{\partial T}{\partial t}, \end{array}$$

where *λ*, *c*_*p*_, and $\dot{q}$ refers to thermal conductivity, specific heat, and metabolic heat source, respectively. Latent heat thus released is considered as ineffective, because insignificant volume ratio of ice to the maximum crystallisable ice *x* is (1 × 10^−3^). Since the diameter of cellular suspension is much smaller than the length of UTS, therefore, heat transfer along the axis can be neglected. Additionally, thermal property within the UTS can be assumed to be uniform and temperature-independent. Thus, the above equation can be simplified as

(2) $$\begin{array}{c} \frac{1}{r}\frac{\partial }{\partial r}\left(\;r\frac{\partial T}{\partial r}\;\right)=\frac{1}{\alpha }\frac{\partial T}{\partial t}, \end{array}$$

where *α* is the thermal diffusivity.

Let the initial temperature of the suspension be *T*_0_ and let the final temperature be *T*_*∞*_. Let the dimensionless form of temperature be expressed as

(3) $$\begin{array}{c} \theta^{*}=\frac{T-T_{\infty }}{T_{0}-T_{\infty }}. \end{array}$$

Therefore, the transient temperature distribution can be written as

(4) $$\begin{array}{c} \theta^{*}=\overset{\infty }{\underset{n=1}{\sum }}C_{n}\exp\left(\;-\zeta_{n}^{2}F_{0}\;\right)J_{0}\left(\;\zeta_{n}\frac{r}{r_{0}}\;\right), \end{array}$$

where

(5) F0=αtr02,Cn=2ζnJ1ζnJ02ζn+J12ζn,

where *J*_0_ and *J*_1_ are Bessel functions and are positive roots of the transcendental equation defined by *ζ*_*n*_:

(6) $$\begin{array}{c} \zeta_{n}\frac{J_{1}\left(\zeta_{n}\right)}{J_{0}\left(\zeta_{n}\right)}=\frac{2hr_{0}}{k}, \end{array}$$

where heat transfer coefficient is denoted by “*h*” and often it evaluates the quality of the cryopreservation system.

The production of volume fraction of ice (*x*) is governed by the equation

(7) $$\begin{array}{c} \frac{dx}{dt}=\kappa a_{1}\left(\;x\;\right)\left(\;T_{m}-T\;\right)\exp\left(\;\frac{-Q}{RT}\;\right), \end{array}$$

where

(8) κ=Lπλ2νTmrf,a1x=x2/31−x,

where *T*_*m*_, *Q*, *R*, *L*, and *r*_*f*_ refer to temperature at the end of the freezing process, activation energy, gas constant, latent heat, and radius of ice when *x* is equal to 1, respectively. The thickness of transition layer between liquid and solid (“mushy zone”) is *λ*, and *n* is the kinematic viscosity.

**Figure 3:**
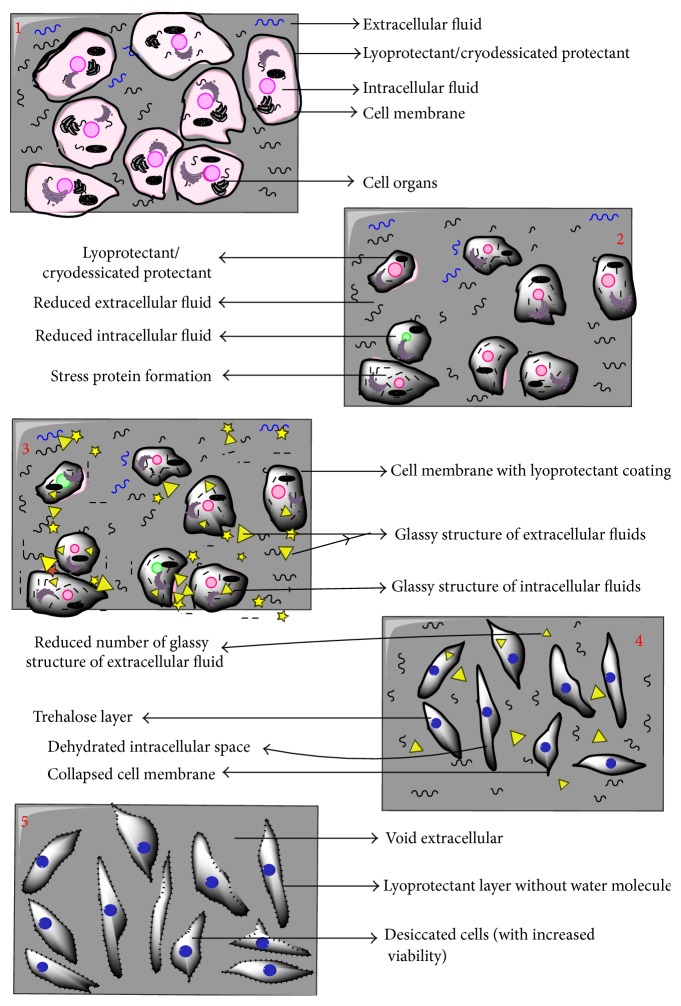
Hypothesized physiological changes being observed in cells following red path for desiccation-lyophilisation phase.

In view of the above thermodynamics describing processes of lyophilisation and desiccation, it has been observed that viability of cell is a vital issue. It results in a requirement of crosstalk between the above two mentioned processes. An approach of crosstalk has also been explained by the three-point thermodynamic diagram. Path defined by red colour as (1→2→3→4→5) is the projected path for the process of desiccation followed by lyophilisation. Sample is at room temperature at point 1; then, it is mildly heated with a gradual decrease in pressure to reach a state of point 2. Point 2 is defined as step-down transition point: slope and intercepts of this point are optimized in order to achieve maximum pressure drop, with minimum increase in temperature and maximum dehydration of the cell. Thus maximum cellular viability can be maintained. Once this state is achieved, the cell mass will be allowed to freezing state while maintaining the temperature to be constant, and point 3 will be reached. Point 3 is a critical point of freezing at low pressure when crystalline state of fluid present in intracellular and extracellular regions will be converted to glassy state. Then, desiccation will be followed by freeze-drying to reach point 4. Since vitrification of cell is done at lower pressure, it might happen that vitrification temperature will be much higher than −80 degrees centigrade. Therefore, pressure drop required to achieve vapour state from freeze state is much higher, reducing the cost of the combined process. Last step is sample dehydration by rapid temperature increase (up to room temperature) at constant pressure to reach point 5. This entire path of crosstalk is able to ensure two vital aspects of preservation of cells: (i) enhancement of cellular viability and (ii) decreasing lyophilisation (freeze-drying) cost. The scheme of hypothesized processes in the cells undergoing desiccation-lyophilisation in accordance with proposed method is depicted in the following (Figure 3).

Cell preservation using crosstalk technique explained in the previous section can be achieved in a controlled chamber. Rate of dehydration of cell can be monitored continuously by a ultrasonic transducer measuring the humidity in the chamber. Cell viability can be continuously monitored by a droplet based remote microscopic system and real-time processing of obtained images. Output of this image-processing system can be used to control temperature and pressure in the chamber.

## 10. Conclusion

Enhanced measures of protection are required for successful anhydrobiotic engineering of MSCs since existing protocols are not able to ensure robust cell recovery. Although the lyophilisation and desiccation are found to be efficient in some cases, the limitations of individual methods impart certain rigidity of their implementation. At the same time, maintenance of the dried cells viability in long-term storage is another critical issue which needs to be addressed. Continuous monitoring and control of preservation process can ensure successful MSCs preservation, but optimization for all the physical parameters is also needed.

## Figures and Tables

**Table 1:** Advantages of trehalose over sucrose as a lyoprotectant.

Sucrose	Trehalose
Vulnerable to hydrolysis under acidic conditions	Not prone to hydrolysis under acidic conditions

Smaller hydration radius; thus large amount of sucrose is required for lyophilisation	Hydration radius 2.5 times that of sucrose, thus requiring 2.5 times less trehalose for lyophilisation as compared to sucrose

Does not protect cells and proteins from oxidative damage	Protects cells and proteins from oxidative damage

Less interaction with water and hence does not displace water molecules bound to carbonyls at the phospholipid bilayer of the cell membrane	Interacts more strongly with water and thus able to displace water molecules bound to carbonyls at the phospholipid bilayer of cell membranes

Lower transition temperature	High transition temperature

**Table 2:** Different methods to deliver trehalose into cells.

Method	Explanation	Reference
Electroporation	Murine myeloma cells were loaded with trehalose by electroporation, then freeze-dried, and rehydrated	[31]

Genetically engineering the cells	Human primary fibroblasts were transfected with otsA and otsB genes from *E. coli*, encoding for trehalose synthase	[32]

Genetically engineered pores	Genetically engineered mutant of alpha-hemolysin from *Staphylococcus aureus *was used to create pores in the cellular membrane of 3T3 fibroblasts and human keratinocytes. Resulting nonselective pore is equipped with a metal-actuated switch that is sensitive to extracellular zinc concentrations, thus permitting controlled loading of trehalose	[33]

Fluid phase endocytosis	Human MSCs were loaded by trehalose up to 30 mM internal concentration at usual cultivation conditions for 24 h at 37°C in the presence of MSC medium with 0–125 mM trehalose	[34]

Endogenous cell surface receptor	TF-1 cells were permeabilized using an endogenous protein P2X7	[35]

Cell penetrating peptides (CPPs)	Trehalose was coupled with CPP and incubated with mouse embryonic fibroblast cells at usual cultivation conditions	[36]
